# Aliovalent Dilute Doping and Nano‐Moiré Fringe Advance the Structural Stability and Thermoelectric Performance in *β*‐Zn_4_Sb_3_

**DOI:** 10.1002/advs.202201802

**Published:** 2022-06-26

**Authors:** I‐Lun Jen, Kuang‐Kuo Wang, Hsin‐Jay Wu

**Affiliations:** ^1^ Department of Materials Science and Engineering National Yang Ming Chiao Tung University Hsinchu 30010 Taiwan; ^2^ Department of Materials and Optoelectronic Science National Sun Yat‐sen University Kaohsiung 80424 Taiwan

**Keywords:** *β*‐Zn_4_Sb_3_ thermoelectrics, aliovalent dilute doping, figure‐of‐merit (zT), nano‐moiré fringes

## Abstract

Thermoelectric (TE) generators have come a long way since the first commercial apparatus launched in the 1950s. Since then, the *β*‐Zn_4_Sb_3_ has manifested its potential as a cost‐effective and environmentally friendly TE generator compared with the tellurium‐bearing TE materials. Although the *β*‐Zn_4_Sb_3_ features an intrinsically low thermal conductivity *κ*, it suffers from a long‐lasting structural instability issue arising from the highly mobile zinc ions. Herein, the dilute Ga dopant gives rise to the aliovalent substitution, lowers the mobile zinc ions, and optimizes the hole carrier concentration *n*_H_ simultaneously. Meanwhile, the formation of nano‐moiré fringes suggests the modulated distribution of point defect that results from soluble Ga in a *β*‐Zn_4_Sb_3_ lattice, which elicits an ultralow lattice thermal conductivity *κ*_L_ = 0.2 W m^−1^ K^−1^ in a (Zn_0.992_Ga_0.008_)_4_Sb_3_ alloy. Hence, a fully dense *β*‐Zn_4_Sb_3_ incorporated with the dilute Ga doping reveals superior structural stability with a peak *zT* > 1.4 at 623 K. In this work, the aliovalent dilute doping coupled with phase diagram engineering optimizes the fluxes of moving electrons and charged ions, which stabilizes the single‐phase *β*‐Zn_4_Sb_3_ while boosting the TE performance at the mid‐temperature region. The synergistic strategies endow the ionic crystals with a thermodynamic route, which opens up a new category for high‐performance and thermal robust TE alloys.

## Graphical abstract

Aliovalent cation substitution between Zn^2+^ and Ga^3+^ diminishes the highly mobile Zn^2+^ and optimizes the hole carriers, leading to an ultrahigh *PF* = 1.6 mW m^−1^ K^−2^. The interstitial Ga^0^ induces the formation of nano‐moiré fringes and thus elicits an ultralow *κ*_L_ = 0.2 W m^−1^ K^−1^. A light‐doped (Zn_0.992_Ga_0.008_)_4_Sb_3_ crystal attains a peak *zT* of 1.4 in the *β*‐Zn_4_Sb_3_ single‐phase region.

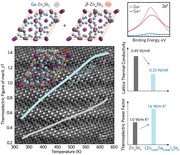

## 1 Introduction

Reducing the usage of fossil fuels realizes the goal of zero carbon footprint by 2035. The need for a sustainable future revitalizes the development of high‐efficiency green technologies that harvest various forms of energy into electricity. In this regard, the thermoelectric (TE) technology, which can turn the undesired waste heat into precious electricity,^[^ ^1^, ^2^ ^]^ emerges as a solution for the energy shortage issue while easing the environmental impact. For a TE alloy, the amount of electricity produced from the thermal energy can be evaluated by the thermoelectric figure‐of‐merit *zT* = (*S* ^2^/*ρκ*)T, which comprises the mutual correlative Seebeck coefficient *S*, the electrical resistivity *ρ*, and the thermal conductivity *κ*.

Since the 1960s, the tellurium‐bearing semiconductors or semimetals have been the mainstream for state‐of‐the‐art TE materials^[^ ^3^, ^4^, ^5^, ^6^, ^7^, ^8^ ^]^ Being one of the exceptions, the *β*‐Zn_4_Sb_3_, which comprises environmental‐friendly and earth‐abundant elements, shows a decent TE performance at a medium temperature.^[^ ^9^ ^]^ Those pros shall make the *β*‐Zn_4_Sb_3_ an ideal TE material, but the mass production of Zn_4_Sb_3_‐based alloys is still on hold. The primary concern arises from the structural instability caused by the highly‐mobile zinc ions and zinc interstitials.^[^ ^10^, ^11^ ^]^ In addition to the electronic conduction, the mobile zinc ions bring the ionic conduction behavior to the *β*‐Zn_4_Sb_3_, which amplifies the phonon‐glass electron‐crystal (PGEC) characteristic and induces the mass transfer caused by the charged ions.^[^ ^12^, ^13^ ^]^ The former effect advances the TE performance, yet the latter harms the thermal robustness, especially when *β*‐Zn_4_Sb_3_‐based alloys are exposed to the external thermal gradient or/and electrical field.^[^ ^14^ ^]^

Given that the grain boundary paves the route for ionic diffusion,^[^ ^15^ ^]^ the *β*‐Zn_4_Sb_3_ with a lower grain boundary density shall retain better structural stability at elevated temperatures. By previous studies, the transport properties of Zn_4_Sb_3_‐based alloys perform varying repeatability for different synthesis routes.^[^ ^16^ ^]^ In this work, the fully dense Zn_4_Sb_3_‐based alloys are synthesized through the Bridgman method. Within the temperature range of 300–625 K, superior crystallinity and improved thermal robustness are obtained in those as‐grown crystals that perform good structural stability.

The subsequent strategy lies in the impurity doping that enables carrier optimization and defect engineering. Per our previous study, the indium (In) dopant lifts the peak *zT* of *β*‐Zn_4_Sb_3_ by enhancing the *PF* while decreasing the *κ*.^[^ ^17^ ^]^ By analogy with the aliovalent doping in the In‐Zn_4_Sb_3_, the gallium (Ga) element shall fulfill the *PF*‐*κ* decoupling. What makes this work unique is the aspect of *dilute* doping that sustains the intrinsic framework of *β*‐Zn_4_Sb_3_. Apart from carrier optimization, the dilute Ga dopant gives rise to the formation of nanoscale defects that elicit an ultralow lattice thermal conductivity *κ*_L_. Nanostructuring comprising the nano‐Moiré fringe, and ZnSb nanoprecipitates not only brings down the *κ*_L_ to the amorphous limit of *β*‐Zn_4_Sb_3_^[^ ^18^, ^19^ ^]^ but enables the filtering effect that raises or retains the *S*.^[^ ^20^ ^]^ In this work, a synergy of dilute aliovalent doping coupled with phase diagram engineering^[^ ^21^ ^]^ and defect engineering^[^ ^22^, ^23^ ^]^ copes with the long‐standing issues in the Zn_4_Sb_3_ based alloys, which could be even exploited to other ionic solids.

## 2 Results and Discussion

**Figure 1:**
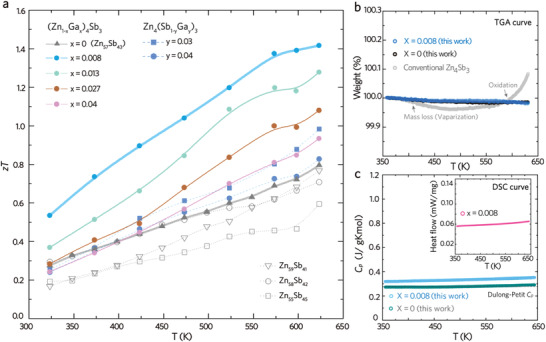
Thermoelectric figure‐of‐merit, composition fluctuation, and phase diagram. a) Temperature‐dependent *zT* values for the three series of Ga‐Zn_4_Sb_3_ alloys and the binary system, which are (Zn_1−_ *_x_* Ga*_x_*)_4_Sb_3_ (*x* = 0, 0.008, 0.013, 0.027, and 0.04), Zn_4_(Sb_1−_ *_y_* Ga*_y_*)_3_ (*y* = 0.03 and 0.04), and Zn_z_Sb_100−z_ (*z* = 55, 58, and 59). b) TGA heating curves for (Zn_1−_ *_x_* Ga*_x_*)_4_Sb_3_ (*x* = 0, 0.008) and conventional Zn_4_Sb_3_ c) Heat capacity curves for (Zn_1−_ *_x_* Ga*_x_*)_4_Sb_3_ (*x* = 0, 0.008). Inset shows the DSC heating curve of (Zn_0.992_Ga_0.008_)_4_Sb_3_.

There has been a long‐lasting debate regarding the intrinsic transport properties, homogeneity region, and high‐temperature stability of single‐phase *β*‐Zn_4_Sb_3_.^[^ ^24^, ^25^ ^]^ We settled this dispute by starting with the analyses of four binary alloys, the Zn_55_Sb_45_, Zn_57_Sb_43_, Zn_58_Sb_42_, and Zn_59_Sb_41,_ which grew by the Bridgman method. As suggested by microstructures, X‐ray diffraction (XRD) patterns (Figure S1, Supporting Information), and wavelength dispersive X‐ray spectroscopy (WDS) analysis (Table S1, Supporting Information), the Zn_57_Sb_43_ reveals the single‐phase feature of *β*‐Zn_4_Sb_3_. By contrast, the Zn‐deficient Zn_55_Sb_45_ contains the ZnSb precipitation, while the Zn‐rich Zn_58_Sb_42_ and Zn_59_Sb_41_ have the Zn as the impurity phase. The slight Zn/Sb ratio variation causes the microstructural variations and induces distinct thermal/electronic transport properties. Figure S2 (Supporting Information) collects the temperature‐dependent *ρ*, *S*, and *κ* curves for Zn_55_Sb_45_, Zn_57_Sb_43_, Zn_58_Sb_42_, and Zn_59_Sb_41_ in the temperature range of 300–650 K. Compared with single‐phase Zn_57_Sb_43_, the Zn‐deficient Zn_55_Sb_45_ exhibits a higher *ρ* curve with a boosted *S* (Figure S2a,b, Supporting Information) which can be ascribed to the reduced *n*_H_ (Table S2, Supporting Information). The Zn_58_Sb_42_ and Zn_59_Sb_41_, on the other hand, show the decreasing *ρ* and *S* curves, implying a higher level of *n*_H_ due to the enrichment in Zn. Figure S2c (Supporting Information) shows that the Zn‐rich Zn_59_Sb_41_ presents the highest *PF* curve among these four binary alloys. Nevertheless, the excess Zn also causes thermal instability at the elevated temperature, as evidenced by a sudden rise in its *PF* curve. Besides the electrical transport properties, the stoichiometric ratio also affects thermal conduction. At the low‐temperature region, the *κ* values drop when the composition shifts from Zn_55_Sb_45_ to Zn_58_Sb_42_ and rapidly increases to *κ* = 1.4 W m^−1^ K^−1^ in the Zn‐excess Zn_59_Sb_41_ alloy (Figure S2d, Supporting Information). The Zn precipitate (Figure S1e, Supporting Information) is responsible for such an increase in the *κ* value. The *κ*_L_ curves (an inset in Figure S2d, Supporting Information) also reveal a decreasing tendency with an increasing Zn/Sb ratio, implying that the excess Zn atoms (e.g., Zn_58_Sb_42_) might induce structural disordering that brings an additional scattering mechanism. However, the *κ*_L_ curve lifts as long as the Zn precipitate becomes significant (e.g., Zn_59_Sb_41_). Consequently, the *zT* peak value (**Figure** **1**a) varies from 0.6 to 0.8 at 623 K as the binary nominal composition shifts from Zn‐deficient Zn_55_Sb_45_ to Zn‐rich Zn_59_Sb_41_. The Zn_57_Sb_43_, however, defines the single‐phase *β*‐Zn_4_Sb_3_ in the binary Zn‐Sb system, as its microstructure and XRD pattern are both free of impurity phases. Given that the single‐phase Zn_57_Sb_43_ performs the best structural stability, this specific composition anchors the starting composition for all the Ga‐Zn_4_Sb_3_ alloys in the following discussion.

The (Zn_1−_ *_x_* Ga*_x_*)_4_Sb_3_ unifies the concepts of aliovalent substitution and dilute impurity doping within the doping range of *x* = 0.008 to *x* = 0.04. Figure 1a shows that the (Zn_1−_ *_x_* Ga*_x_*)_4_Sb_3_ alloys reveal the uprising *zT* curves compared with the undoped *x* = 0. The lightly doped *x* = 0.008 has an outperforming *zT* > 1.4 in the temperature range of 523–623 K. The high‐*zT* plateau makes the lightly doped *x* = 0.008 endure a large temperature difference (Δ*T*) between the hot side and cold side when utilized into a TE device. In addition to the aliovalent cation substitution, another off‐stoichiometric Ga‐Zn_4_Sb_3_ was prepared in accordance with an excess cations condition. Nevertheless, the *y*‐series Zn_4_(Sb_1−_ *_y_* Ga*_y_*)_3_ (*y* = 0.03 and 0.04) show the descending trend in peak *zT* values with increasing *y*, suggesting that the excess cations might degrade the TE performance of Zn_4_Sb_3_‐based alloys.

Apart from the high *zT* values, the as‐grown *x*‐series samples perform good thermal and structural stability as proved by thermogravimetric analysis (TGA) and differential scanning calorimetry (DSC) analysis (Figure 1b,c). The TGA curves (Figure 1b) of *x* = 0 and *x* = 0.008 reveal no noticeable mass loss below 650 K, while the DSC curve (inset of Figure 1c) further suggests that the *x* = 0.008 is free of phase transition in the temperature range of 350–650 K. Moreover, the heat capacity *C*_p_ of *x* = 0 and *x* = 0.008 follows the estimation of Dulong–Petit law,^[^ ^26^, ^27^ ^]^ implying that the dilute impurity doping (e.g., *x* = 0.008) does not change the intrinsic property of *β*‐Zn_4_Sb_3_. By contrast, one piece of commercial Zn_4_Sb_3_ bulk fails to retain thermal stability. Its TGA curve (gray curve in Figure 1b) shows apparent mass loss and severe oxidation, suggesting that the commercial Zn_4_Sb_3_‐based materials are unstable at elevated temperatures.

**Figure 2:**
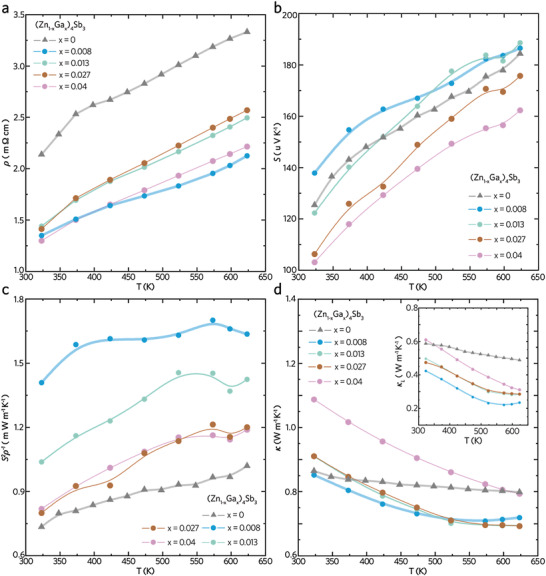
Thermoelectric figure‐of‐merit, composition fluctuation, and phase diagram. a) Temperature‐dependent electrical resistivity *ρ* for the three series of Ga‐Zn_4_Sb_3_ alloys, which are (Zn_1−_ *_x_* Ga*_x_*)_4_Sb_3_ (*x* = 0, 0.008, 0.013, 0.027, and 0.04). b) Seebeck coefficient *S*, c) power factor *S* ^2^ *ρ* ^−1^, and d) thermal conductivity *κ* from 320 to 623 K. Inset shows lattice thermal conductivity.

As presented in Figure 1a, the *zT* curves of the *x*‐ and *y*‐series alloys span widely, despite their compositions showing minor variations. For both series of alloys, the incorporation of Ga significantly brings down the *ρ* curves (**Figure** **2**a and Figure S3a, Supporting Information) by increasing the *n*_H_ (Table S2, Supporting Information). Hence, those Ga‐Zn_4_Sb_3_ alloys reveal the boosted *PF* values (Figure 2c and Figure S3c, Supporting Information) compared with the undoped sample. In particular, the *S* curves of lightly doped *x* = 0.008 and *x* = 0.013 alloys are slightly lifted (Figure 2b and Figure S3b, Supporting Information), revealing the *ρ*–*S* decoupling that yields a high‐rise *PF* curve in the best‐performing *x* = 0.008. A peak *PF* value of 1.65 mW m^−1^ K^−1^ is achieved in the lightly doped *x* = 0.008, showing a 150% enhancement compared with the undoped *x* = 0 (Figure 2c). Such *ρ*–*S* decoupling has been found in high‐performance In‐Zn_4_Sb_3_ alloys,^[^ ^17^, ^28^ ^]^ attributed to the energy filtering effect^[^ ^29^, ^30^ ^]^ arising from the well‐distributed nanoprecipitates (e.g., InSb in In‐Zn_4_Sb_3_). The nanoprecipitates introduce a high density of internal boundaries that scatter the long‐wavelength phonons without impeding the electron transportation. Meanwhile, the high‐density interfaces between the nanoprecipitates and the matrix form the energy barriers blocking low‐energy carriers. Only the high‐energy carriers could overpass the barriers and traverse the crystalline matrix. The energy filtering effect gives rise to the boosted thermopower *S* even under the increasing *n*_H_, which explains the *ρ*–*S* decoupling in our best‐performing Ga‐Zn_4_Sb_3_. Hence, the formation of nanoscale heterostructure could be expected in the *x* = 0.008. By contrast, the *PF* curves for *y*‐series alloys (Figure S3c, Supporting Information) fall in a moderate region due to the counterbalance between *ρ* and *S*.

The *κ* curves also play a vital role in the resultant TE performance. At elevated temperatures, the *x*‐series alloys show suppressed *κ* curves, yet the *y*‐series alloys present higher *κ* curves than the undoped sample (Figure 2d and Figure S3d, Supporting Information). Note that the *κ*_L_ curve of best‐performing *x* = 0.008 remains low‐lying in the whole measurement window and reaches an exceptionally low value of 0.2 W m^−1^ K^−1^ (inset of Figure 2d). Such an ultralow *κ*_L_ even breaks the amorphous limit of intrinsic *β*‐Zn_4_Sb_3_, suggesting that the dilute Ga doping introduces an additional scattering mechanism.

**Figure 3:**
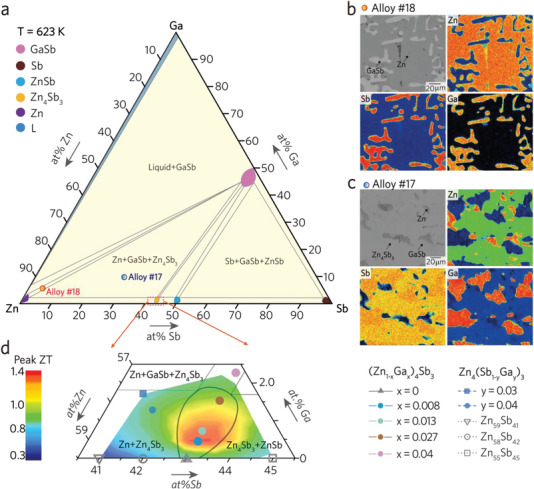
a) Isothermal section of Zn‐Sb‐Ga at 623 K superimposed with nominal compositions of thermally equilibrated alloys. BEI micrographs of Zn‐Sb‐Ga alloys equilibrated for over 60 d at 623 K: b) alloy #18 (Zn‐5.0 at%Sb‐5.0 at%Ga), c) alloy #17 (Zn‐30.0 at%Sb‐10.0at%Ga). Note that the wavelength dispersive X‐ray spectroscopy (WDS) mapping for individual elements of Zn (upper‐right image), Ga (lower‐right image), and Sb (lower‐left) for alloy #18 and #17 were obtained by field‐emission EPMA. d) Magnified Zn‐Sb‐Ga isothermal section at 623 K superimposed with the color contour of peak *zT* values at 623 K.

As aforementioned, Ga is an effective dopant to improve thermal/structural stability while simultaneously boosting the TE performance. The optimal compositional window is restricted to a dilute doping region as the light‐doped Ga‐Zn_4_Sb_3_ alloys perform best. This work pinpoints a high‐*zT* zone by superimposing the transport properties on the equilibrium phase diagram. Such a phase diagram engineering has been exploited into several state‐of‐the‐art TE alloys, including the In‐Zn_4_Sb_3_,^[^ ^17^ ^]^ Sb‐GeTe,^[^ ^31^ ^]^ Cu‐Bi_2_Te_3_,^[^ ^32^ ^]^ etc., leading the tremendous success in *zT* enhancement. Herein, we optimize the TE performance of Ga‐Zn_4_Sb_3_ via a similar track. Various ternary Ga‐Zn‐Sb alloys with predetermined compositions were post‐annealed at 623 K for two months (Table S3, Supporting Information). Their equilibrium phase compositions, microstructures, and XRD patterns were collected to construct the Ga‐Zn‐Sb phase diagram (**Figure** **3**a).

Two thermally equilibrated alloy #18 (Zn‐5.0 at%Sb‐5.0 at%Ga) and alloy #17 (Zn‐30.0 at%Sb‐10.0 at%Ga) are taken as examples revealing the procedures of phase diagram determination. The microstructures, together with the WDS elemental mapping (Figure 3b,c) and XRD patterns (Figure S4a,b, Supporting Information), confirm the existence of two‐phase GaSb+Zn (alloy #18) and three‐phase *β*‐Zn_4_Sb_3_+GaSb+Zn (alloy #17). The equilibrium information could be collected (Figure S5, Supporting Information). The phase diagram recognizes that the single‐phase *β*‐Zn_4_Sb_3_ has an asymmetric homogeneity with a maximal solubility of 2 at%Ga. The superposition of the magnified phase diagram and peak *zT* values yields the *zT* mapping, emphasizing a high‐*zT* zone by color contours (Figure 3d). The *x*‐series alloys locate inside the homogeneous *β*‐Zn_4_Sb_3_, while the *y*‐series alloys fall out of *β*‐Zn_4_Sb_3_ after lightly doping. The magnified phase diagram suggests that the *x*‐series alloys mainly retain the single‐phase *β*‐Zn_4_Sb_3_ while the *y*‐series alloys fall in the *β*‐Zn_4_Sb_3_+Zn two‐phase region. That distinct phase features explain their varying TE performance.

Although the phase diagram engineering has shown the strength in boosting the TE performance,^[^ ^21^ ^]^ some assumptions need to be drawn before the thermodynamic approach could be realistically used. First, it is assumed that the diffusion‐controlled growth elicits the local equilibrium at the interface between liquid melt and crystal solid during the Bridgman growth. Second, the Bridgman method allows a liquid melt to solidify at a slower pace, and it minimizes the evaporation of the Zn atom as well as maximizes the dopant solubility.^[^ ^28^ ^]^ Under those circumstances, an equilibrium phase diagram can forecast the phase feature for an alloy grown by the Bridgman method. As evident by the powder XRD patterns (Figure S6, Supporting Information), most of the as‐grown *x*‐series alloys are of single‐phase *β*‐Zn_4_Sb_3_ with high relative densities *R* > 98% (Table S2, Supporting Information), which are consistent with that predicted by the phase diagram. The Rietveld refinement of the powder XRD patterns of single‐phase alloys suggests an increasing trend in lattice constants *a* and *c* to the growing *x* (Figure S7, Supporting Information). The isotropically expanded *β*‐Zn_4_Sb_3_ lattice shall be attributed to the interstitial bulky Ga^0^ (187 pm), as the substitution of larger Zn^2+^ (74 pm) by smaller cation Ga^3+^ (62 pm) controdictionally leads to lattice shrinkage. Hence, soluble Ga possibly presents the mixing chemical states of elemental Ga^0^ and cation Ga^3+^, which will be proved by the following X‐ray photoelectron spectroscopy (XPS) analysis.

One emerging question is the origin of elemental Ga^0^ in the *β*‐Zn_4_Sb_3_ lattice. The possible answer is the nonequilibrium freezing during the Bridgman growth that yields the microsegregation and nanoscale inhomogeneity for solute distribution. Table S3 (Supporting Information) shows the Ga solubility in the Zn_4_Sb_3_ from the thermally equilibrated Ga‐Zn_4_Sb_3_ alloys, which reaches 3–4 at%. By contrast, the as‐grown *x*‐series alloys exhibit a lower Ga concentration in the Zn_4_Sb_3_ phase (Table S1, Supporting Information). The deviation in Ga solubility can be explained by the Scheil model (the nonequilibrium lever rule).^[^ ^33^ ^]^ During the nonequilibrium freezing, the liquid melt is enriched with the solute (Ga in our case), making the solidified solid contains less solute. When the freezing is complete, the Ga‐enriched liquid forms the Ga‐rich Ga‐Zn_4_Sb_3_, embedding in the Ga‐deficient Zn_4_Sb_3_. This microsegregation will be proved in the following transmission electron microscopy (TEM) analysis.

**Figure 4:**
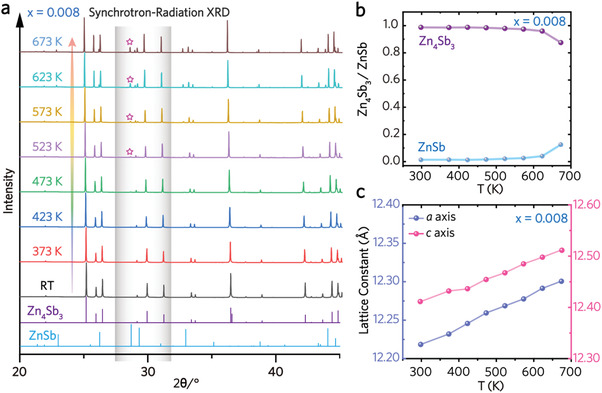
a) Synchrotron‐radiation XRD patterns of (Zn_0.992_Ga_0.008_)_4_Sb_3_ from room temperature to 720 K, b) molar fraction (Zn_4_Sb_3_ and ZnSb) of (Zn_0.992_Ga_0.008_)_4_Sb_3_ are calculated by in situ XRD results, and c) lattice constants (*a‐* and *c*‐axis) of (Zn_0.992_Ga_0.008_)_4_Sb_3_ in the temperature range of 300–673 K.

An in situ synchrotron‐radiation diffractometer is performed on the best‐performing *x* = 0.008 to uncover the temperature‐dependent structural transition. **Figure** **4**a shows the high‐temperature synchrotron‐radiation XRD patterns in the temperature range of 300–673 K. The characteristic peaks of ZnSb (2*θ*° = 26.20°, 28.67°, 29.30°) emerge when the temperature is above 523 K, which grow stronger with increasing temperature. The calculated phase ratio of *β*‐Zn_4_Sb_3_/ZnSb (Figure 4b) implies that the *β*‐Zn_4_Sb_3_ retains the majority phase when the temperature is lower than 623 K. When the temperature elevates above 623 K, the contribution of ZnSb cannot be neglected. Hence, the TE property assessment for Zn_4_Sb_3_‐based alloys shall be conducted below 623 K to ensure that the acquisitions reflect the intrinsic property of *β*‐Zn_4_Sb_3_. Moreover, the lattice volume of *β*‐Zn_4_Sb_3_ expands isotropically with the increasing temperature, as the lattice constants *a* and *c* show a linear relationship with temperature (Figure 4c).

**Figure 5:**
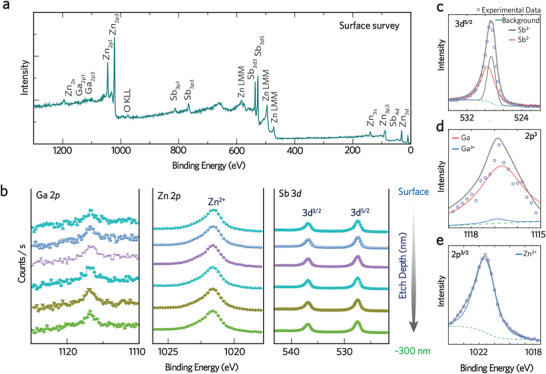
a) XPS surface survey spectrum analyses of *x* = 0.008, b) evolution of the Ga 2p, Zn 2p, Sn 3d core‐level spectra as a function of etch depth. The XPS depth profile analyses show the innermost layer of c) Sb 3d^5/2^, d) Ga 2p^3^, and e) Zn 2p^3^. The blue open circles show the raw photoelectron data, while the gray curve was drawn to guide the eye.

Given that the light impurity doping boosts the TE performance of Zn_4_Sb_3_‐based alloys, the role of the dilute dopant becomes vital. The XPS analysis validates the chemical states of constituent elements in the *x* = 0.008 sample. The surface survey spectrum (**Figure** **5**a) identifies the contributions of Zn 2p and Sb 3d and reveals a trace amount of Ga 2*p* in the Zn_4_Sb_3_. The XPS profile of Zn 2p, Sb 3d, and Ga 2p was acquired from the surface to an etch depth of 300 nm in one *x* = 0.008 sample (Figure 5b). Note that two broadening peaks at 527 and 536 eV appertain to the binding energy of Sb 3d_5/2_ and Sb 3d_3/2_. The peak at 527 eV can be further decoupled to prove the mixing contributions from Sb^−2^ at 527.8 eV and Sb^−3^ at 527.4 eV (Figure 5c), suggesting that the Sb atoms are stabilized in a mixing valence of −2 and −3.^[^ ^34^ ^]^ In the Ga 2p binding energy diagram (Figure 5d), a peak located at a binding energy of 1116.8 eV also infers the coexistence of elemental Ga^0^ (binding energy of 1116.9 eV) and Ga^3+^ (binding energy of 1116.7 eV). Likewise, the Zn 2p diagram (Figure 5e), which presents a peak at 1022 eV, verifies the contribution of ionic Zn^2+^.^[^ ^35^ ^]^

The XPS analysis concludes that dilute Ga has the mixing chemical states (Ga^0^ and Ga^3+^) and acts as an aliovalent dopant in the Zn_4_Sb_3_‐based alloys. The element Ga^0^ and the cation Ga^3+^ play different roles in manipulating the electrical and thermal transport properties. The elemental Ga^0^ forms as an interstitial solute and simultaneously expands the Zn_4_Sb_3_ lattice (Figure 4c) and Figure S7, Supporting Information), introducing the point defects that enhance the phonon scattering. On the other side, the substitution of mobile Zn^2+^ ions by Ga^3+^ suppresses the ionic conduction of Zn_4_Sb_3_, leading to the decreasing carrier mobility *μ*_H_ with the increasing Ga content (Table S2, Supporting Information). Meanwhile, the aliovalent substitution of Zn^2+^ by Ga^3+^ tunes the *n*_H_ (Table S2, Supporting Information), enhancing the electrical conductivity *σ* = *ρ* ^−1^. The III‐A group realizes the aliovalent doping strategy that optimizes ionic and electronic conduction contribution in the Zn_4_Sb_3_. The suppressing ionic conduction improves the structural stability while the aliovalent Ga^3+^ amplifies the electronic p‐type conduction, which synergistically boosts the *PF*. Last but not least, the existence of elemental Ga^0^ acts as the source of point defect that simultaneously lowers the *κ*_L_.

**Figure 6:**
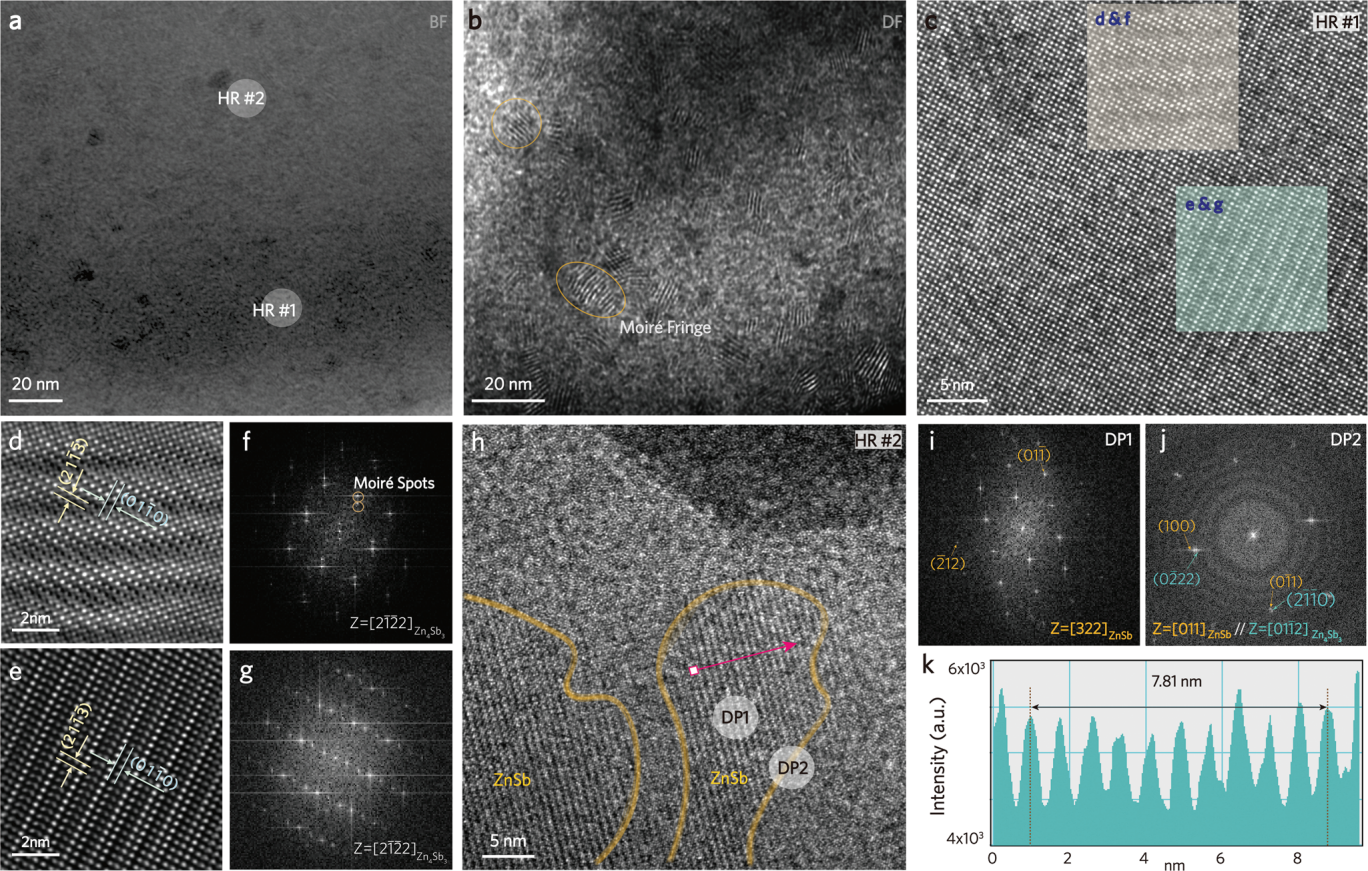
TEM analyses of (Zn_0.992_Ga_0.008_)_4_Sb_3_ (*x* = 0.008) including the a) bright‐field (BF) image and b) dark‐field (DF) image. c–e) HRTEM image with sample#1. f,g) Diffraction pattern. h) HRTEM image with sample#2. i,j) Diffraction pattern of single‐phase ZnSb and mixed phases ZnSb+Zn_4_Sb_3_. k) Line profile of the ZnSb phase.

Apart from being a structural stabilizer and carrier optimizer, dilute Ga doping fulfills the defect engineering that brings down the *κ*_L_ significantly (inset of Figure 2d). A TEM analysis upon the best‐performing *x* = 0.008 alloy uncovers the nanoscale defects. The low‐magnification bright‐field (BF) image (**Figure** **6**a) suggests a nearly single‐phase feature except for some dark inclusions. Nevertheless, the dark‐field (DF) image indicates the existence of localized lattice imperfection and compositional modulation induced by soluble Ga. The lattice mismatch between the matrix *β*‐Zn_4_Sb_3_ and the Ga‐incorporated *β*‐Zn_4_Sb_3_ gives rise to the formation of moiré fringe (Figure 6b). The scanning TEM (STEM) image and STEM‐EDS mapping also confirm the localized inhomogeneity originated from the modulated Ga distribution (Figure S8, Supporting Information).

A closer observation upon the region containing the moiré fringe yields the high‐resolution images HR #1 (Figure 6c) and two enlarged atomic images (Figure 6d,e). Figure 6d magnifies the region of rotation moiré fringe resulting from the superposition of *β*‐Zn_4_Sb_3_ and Ga‐incorporated *β*‐Zn_4_Sb_3_. On the contrary, Figure 6e confirms the matrix consisting of defect‐free *β*‐Zn_4_Sb_3_. The corresponding diffraction patterns (DP, Figure 6f,g) present the diffraction spots from the hexagonal structure with an identical ${\left[2\bar{1}\bar{1}2\right]}_{Zn_{4}Sb_{3}}$ zone axis. However, the DP of the moiré fringe reveals obvious spot‐splitting (denoted as moiré spots in Figure 6f), suggesting dual hexagonal structures with different interplanar spacings exist. It is speculated that the Ga atoms randomly distribute in some of the *β*‐Zn_4_Sb_3_ lattices, causing the lattice distortion and widening the interplanar spacing. In addition to the moiré fringe, the HR #2 from Figure 6a affirms the existence of ZnSb nanoprecipitation. Recalled from the in situ XRD patterns, a small amount of ZnSb emerges when the temperature is higher than 523 K. The TEM sample reflected the details for one *x* = 0.008 sample after high‐temperature TE measurement. Therefore it was not surprising that the ZnSb nano precipitates were captured in the TEM analysis (Figure 6h). Two DPs, denoted as DP1 and DP2 in Figure 6h, confirm the existence of the impurity phase. The diffraction spots in Figure 6i can be indexed as the orthorhombic structure with a zone axis of [322]_*ZnSb*_, while that in Figure 6j reveals the coexistence of ZnSb and *β*‐Zn_4_Sb_3_ orientating along a zone axis of ${\left[01\bar{1}2\right]}_{Zn_{4}Sb_{3}}//{\left[011\right]}_{ZnSb}$. Finally, the line profile (Figure 6k), which is acquired inside the ZnSb nanoprecipitate, indicates the contribution of (010)_ZnSb_.

**Figure 7:**
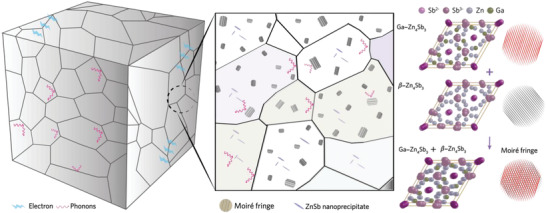
A schematic showing the enhanced phonon scattering in a lightly doped Ga‐Zn_4_Sb_3_. The formation of ZnSb nanoprecipitates and moiré fringes is responsible for its ultralow lattice thermal conductivity.

The threads of evidence imply that the Zn_4_Sb_3_‐based alloys could be cost‐effective and environmentally friendly alternatives to lead tellurides. In this work, the dilute III‐A element doping (for example, Ga) overcomes the long‐lasting hurdles by synergizing structural stabilization, carrier optimization, and *κ*_L_ reduction. The fully dense Zn_4_Sb_3_ crystal grown by the Bridgeman method shows better crystallinity compared with the polycrystalline one. The electrons experience less impedance when traveling through a fully dense crystal than a polycrystal. Moreover, the Bridgman‐grown samples possess a lower concentration of vacancy, and hence the hopping of mobile Zn ions shall be suppressed. The above unveils part of the factors yielding our outperforming and highly stable Ga‐Zn_4_Sb_3_ crystals compared with the state‐of‐the‐art zinc antimonide. In the aspect of *κ*_L_ reduction, a schematic illustration elucidates the impact of dilute Ga doping on thermal conduction (**Figure** **7**). Dilute Ga doping not only induces the elemental Ga^0^ as point defects but elicits the uniform distribution of moiré fringe with a feature size of tens of nanometer. Those defects concertedly scatter the short‐to‐mid wavelength phonons. Meanwhile, the small amount of ZnSb nanoprecipitate and grain boundaries impedes the mid‐to‐long wavelength phonons. Hence, the dilute Ga doping realizes the defect engineering and leads to the ultralow *κ*_L_ < 0.3 W m^−1^ K^−1^ at 623 K in the best‐performing *x* = 0.08 alloy.

## 3 Conclusion

In summary, the dilute IIIA‐group Ga doping coupled with phase diagram engineering tackles the long‐lasting structural instability issue in the zinc antimonide, making the high performance and thermally robust Ga‐Zn_4_Sb_3_ alloys possible. Incorporated Ga shows mixed chemical states of Ga^0^ and Ga^3+^, implying the coexistence of interstitial Ga elements and substitutional Ga ions in a *β*‐Zn_4_Sb_3_ lattice. The Ga ions realize the carrier optimization and suppress the ionic conduction originating from the highly‐mobile Zn ions, leading to the high *PF* = *S* ^2^ *ρ* ^−1^ with improved structural stability. In addition, the lightly‐doped *x* = 0.008 reveals the localized compositional fluctuations comprising the Ga‐soluble and Ga‐free *β*‐Zn_4_Sb_3_ lattices. That gives rise to the dense distribution of nanoscale moiré fringes, which greatly reduces the *κ*_L_ by half compared with undoped *x* = 0. As a consequence, the best‐performing and fully dense *x* = 0.008 achieves a high peak *zT* of 1.4 at 623 K, resulting from its high‐rise *PF* = 1.6 mW m^−1^ K^−2^ and the ultralow *κ*_L_ < 0.3 W m^−1^ K^−1^. The synergy of light impurity doping and defect engineering enlightens the tellurium‐free *β*‐Zn_4_Sb_3_ as promising mid‐temperature TE alloys that reconcile the debates between cost, sustainability, and TE performance.

## 4 Experimental Section

### Phase Diagram Determination

High‐purity elements of Zn (99.99%), Sb (99.99%), and Ga (99.99%) were weighed according to the predetermined nominal composition (Table S3, Supporting Information), sealed in the evacuated quartz tubes (≈10^−5^ torr), heated to 1023 K in 5 h, and homogenized at 1023 K for another 2 h. The liquid melts were water‐quenched and subjected to the post‐annealing at 623 K for 45 d. The thermally equilibrated alloys were then cooled down to room temperature by water quenching for the following metallographic observation and structural identification.

### Bridgman Growth for TE Samples

Four series of Ga‐incorporated Zn_4_Sb_3_, the Zn*_x_* (Ga_1−_ *_x_*)_4_Sb_3_, Zn_4_(Sb_1−_ *_y_* Ga*_y_*)_3_, and binary Zn*_Z_* Sb_100−_ *_Z_*, whereas *x* = 0–0.04, *y* = 0.03–0.04, and *z* = 55, 58–59, were synthesized by using the Bridgman method. Starting with the high‐purity elements of Zn (99.99%), Sb (99.99%), and Ga (99.99%), the alloys were premelted at 1023 K for 2 h, followed by a water‐quenching process. The sample ampoules were placed in a Bridgman furnace at a high‐temperature region (*T* = 863 K) where the alloys became liquid melts and moved downward to a low temperature (*T* = 753 K) with a constant growth rate of 3.7 mm h^−1^. The as‐grown samples were cut into pellets and cylinders for TE property measurements.

### TE Properties Measurement

The electrical resistivity *ρ* and Seebeck coefficient *S* were measured by a commercial instrument (ULVAC ZEM‐3) under the helium‐filled atmosphere. The thermal conductivity *κ* comprised three factors according to the equation = *D* × *C_p_* × *d* . The thermal diffusivity *D* was measured by a laser flash method in a commercial instrument (Netzsch LFA 467), the specific heat capacity *C*_p_ was estimated from Dulong–Petit law (*C*_p_ = 3R/M), while the density *d* was obtained by Archimedes method (Chrom Tech JA‐2003J), respectively. The lattice thermal conductivity could be estimated by *κ*_L_ = *κ* − *κ*_e_, where the electronic thermal conductivity *κ*_e_ was calculated using the Wiedemann Franz law* κ_e_* = *LTρ* ^−1^. Note that the Lorentz factor *L* correlates with the *S* via the equation $L\;=\left\{1.5+\exp[-\frac{\left|S\right|}{116}]\right\}\;\times 10^{-8}{\;V}^{2}K^{-2}$.^[^ ^36^ ^]^ The carrier concentration (*n*_H_) and mobility (*μ*_H_) were obtained by Hall measurement (HMS‐3000) under the magnetic field of 0.49T. Thermal analysis included the weight loss assessment, the experimental *C*_p_, and the phase transition temperature determination was conducted on the conventional Zn_4_Sb_3_, the as‐growth Zn_4_Sb_3_, and the (Zn_0.992_Ga_0.008_)_4_Sb_3_, by using the DSC (Netzsch DSC 3500 sirius) and TGA (Mettler‐Toledo), respectively.

### Characterization

For metallographic observation, the thermally equilibrated alloys were mounted in the epoxy resins and ground by a series of SiC papers, ranging from #400 to #4000, and were polished by the Al_2_O_3_ powders with particle sizes from 0.1 to 0.05 µm. A field‐emission probe microanalyzer determined the microstructures and the compositions of different phases (JEOL JXA‐8530F, EPMA). The crystal structures were analyzed by using an in‐house powder X‐ray diffraction (Bruker D2‐Phaser, Germany) with Cu K*α* radiation (*λ* = 1.5406 Å) and an in situ synchrotron‐radiation PXRD at the TPS‐19A beamline of the National Synchrotron Radiation Research Center (NSRRC) in Taiwan, with the wavelength of 0.77489 Å (16 keV). The lattice parameters for the highest *zT* sample (Zn_0.992_Ga_0.008_)_4_Sb_3_ were calculated from the in situ XRD pattern using the interplanar spacing for the hexagonal structure.^[^ ^37^ ^]^ Electron spectroscopy further analyzed the best‐performing sample for chemical analysis (ULVAC PHI 5000 Versaprobe II, ESCA). The surface survey and depth profiles for constitute elements were collected with an achromatic Al K*α* radiation under a sputtering rate of 5.9 nm min^−1^. The nanoscale features of (Zn_0.992_Ga_0.008_)_4_Sb_3_ were further analyzed by field‐emission transmission electron microscopy (FEI E.O Tecnai F20 G2) equipped with an EDS detector, to obtain the high‐resolution image, STEM analysis, EDS analysis, selected area electron diffraction (SAED) patterns, bright‐field image, and dark‐field image.

## Conflict of Interest

The authors declare no conflict of interest.

## Author Contributions

I.‐L.J. synthesized the samples and conducted the sample characterizations and thermoelectric properties measurements. K.‐K.W. conducted the TEM analyses. I.‐L.J. and H.J.W. contributed to the discussion and writing of the manuscript.

## Supporting information

Supporting Information

## Data Availability

The data that support the findings of this study are available in the supplementary material of this article.
