# Supplementary material for: Aliovalent Dilute Doping and Nano‐Moiré Fringe Advance the Structural Stability and Thermoelectric Performance in β‐Zn4Sb3
Source: Adv Sci (Weinh). 2022 Jun 26;9(26):2201802. doi: 10.1002/advs.202201802 (PMC9475506; doi:10.1002/advs.202201802)
Supplement: Supplementary file 1 — Supporting Information [file ADVS-9-2201802-s001.pdf]

ADVANCED SCIENCE
Open Access

Supporting Information

for *Adv. Sci.*, DOI 10.1002/advs.202201802

Aliovalent Dilute Doping and Nano-Moiré Fringe Advance the Structural Stability and Thermoelectric Performance in β-Zn_4Sb_3

*I-Lun Jen, Kuang-Kuo Wang and Hsin-Jay Wu**

Supporting Information

Aliovalent Dilute Doping and Nano-Moiré Fringe Advance the Structural Stability and Thermoelectric Performance in *β*-Zn_4Sb_3

*I-Lun Jen, Kuang-Kuo Wang and Hsin-Jay Wu**

Supplementary Text

Phase diagram determinations

Figure 3a shows the isothermal section of ternary Zn-Sb-Ga at 623 K superimposed with the nominal compositions of thermally-equilibrated alloys. The nominal compositions of thirty-four ternary alloys and the compositions of the equilibrium phases are summarized in **Table S3**. The XRD patterns for selective thermally-equilibrated alloy #18 (two-phase region GaSb + L(Zn,Ga)), alloy #17 (three-phase region Zn_4Sb_3 + GaSb + Zn), alloy #8(two-phase region GaSb + L(Zn,Ga)), alloy #26 (three-phase region Zn_4Sb_3 + GaSb + ZnSb), alloy #29 (two-phase region GaSb + ZnSb), alloy #32 (three-phase region Sb + GaSb + ZnSb) are summarized in **Figure S4**a-b and **Figure S5**a-d, respectively. Taking alloy #18 as an example (**Figure 3**c), the elemental mapping of Ga and Sb reveals three different color contrasts, suggesting the existence of two different equilibrium phases. Based on an XRD pattern (**Figure S4**a), alloy#18 locates in a two-phase region confined by the GaSb and the liquid phase. Another crucial example is alloy #17 (**Figure 2**c), whose nominal composition falls in the three-phase Zn_4Sb_3 + GaSb + Zn.

Characterization

Figure S1 suggests that the ZnSb precipitate is observed in the binary alloy $Zn_{55}Sb_{45}$ while the Zn precipitate is found in $Zn_{58}Sb_{42}$ and $Zn_{59}Sb_{41}$. Both the XRD patterns (**Figure S1**a) and BEI images (**Figure S1**b-e) confirm the transition in phase regions from the two-phase ZnSb + Zn_4Sb_3 ($Zn_{55}Sb_{45}$), to the single-phase Zn_4Sb_3 ($Zn_{57}Sb_{43}$), and finally to the two-phase Zn + Zn_4Sb_3 ($Zn_{58}Sb_{42}$ and $Zn_{59}Sb_{41}$). Furthermore, the **Figure S6**b-e shows the microstructure images for $(Zn_{1-x}Ga_x)_4Sb_3$ (x = 0.008, 0.013, 0.027 and 0.04), respectively. With increasing Ga substitution, the cracks and pores become noticeable. The STEM analysis is conducted on the best-performing x = 0.008. **Figure S8**a-d reveals the STEM image and the corresponding elemental mapping of Zn, Ga, and Sb. The STEM image shows the localized compositional inhomogeneity that could originate from the Ga element's modulated distribution. Additionally, the secondary phase GaSb is observed in x = 0.04 (**Figure S6**e), which increases the electrical resistivity and lattice thermal conductivity (**Figure 2**a and d). However, **Figure S9** shows the XRD patterns and BEI images for $Zn_4(Sb_{1-y}Gay)_3$ (y = 0.03 and 0.04). With the increasing y, the amount of Zn precipitation increases (**Figure S9**b-c), affecting the electrical resistivity decreased.

Thermoelectric property

Figure S2a-d collects the TE properties for the above-mentioned binary alloys in the temperature range of 300 K to 650 K. The decreasing Zn ratio lifts both the ρ and S values, owing to the formation of ZnSb phase. **Figure S3**a-d shows the temperature-dependent ρ, S, $S^2\rho^{-1}$, and κ of $Zn_4(Sb_{1-y}Ga_y)_3$ (y = 0.03 and 0.04). Compared with the undoped x = 0, the y-series alloys have higher $S^2\rho^{-1}$ values. However, the κ curves (**Figure S3**d) is significantly lifted, hence degrading their zT values.

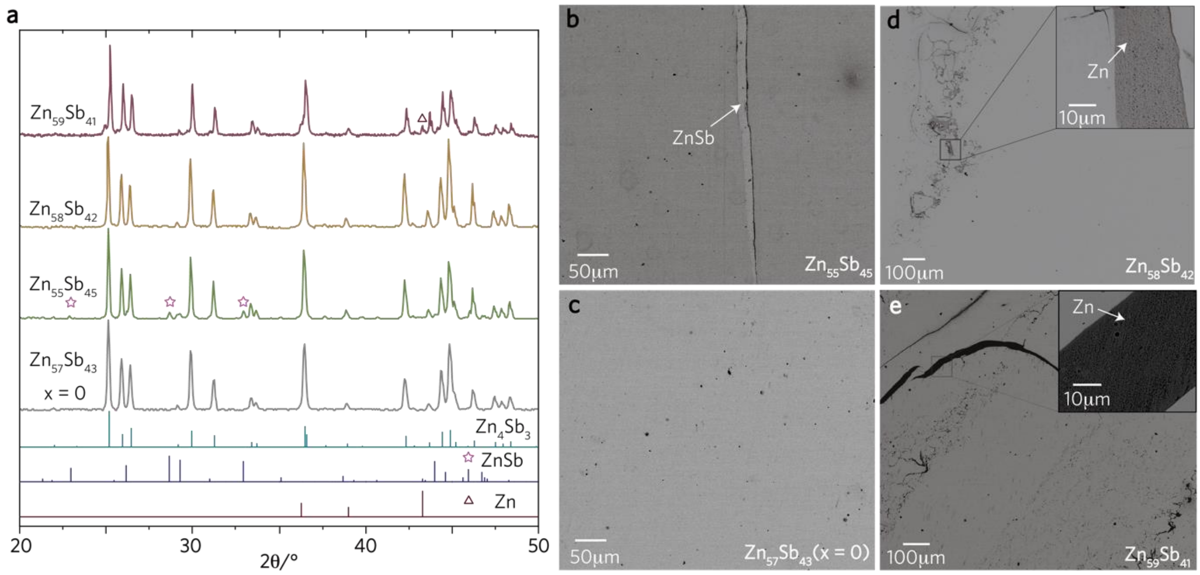

Figure S1. a) XRD patterns of Ga-Zn_4Sb_3 TE alloy for Zn_4Sb_3 and Zn_zSb_{100-z} (z = 59, 58, and 55), the backscattered images of binary Zn-Sb TE alloy: b) $Zn_{55}Sb_{45}$, c) Zn_4Sb_3, d) $Zn_{58}Sb_{42}$, e) $Zn_{59}Sb_{41}$.

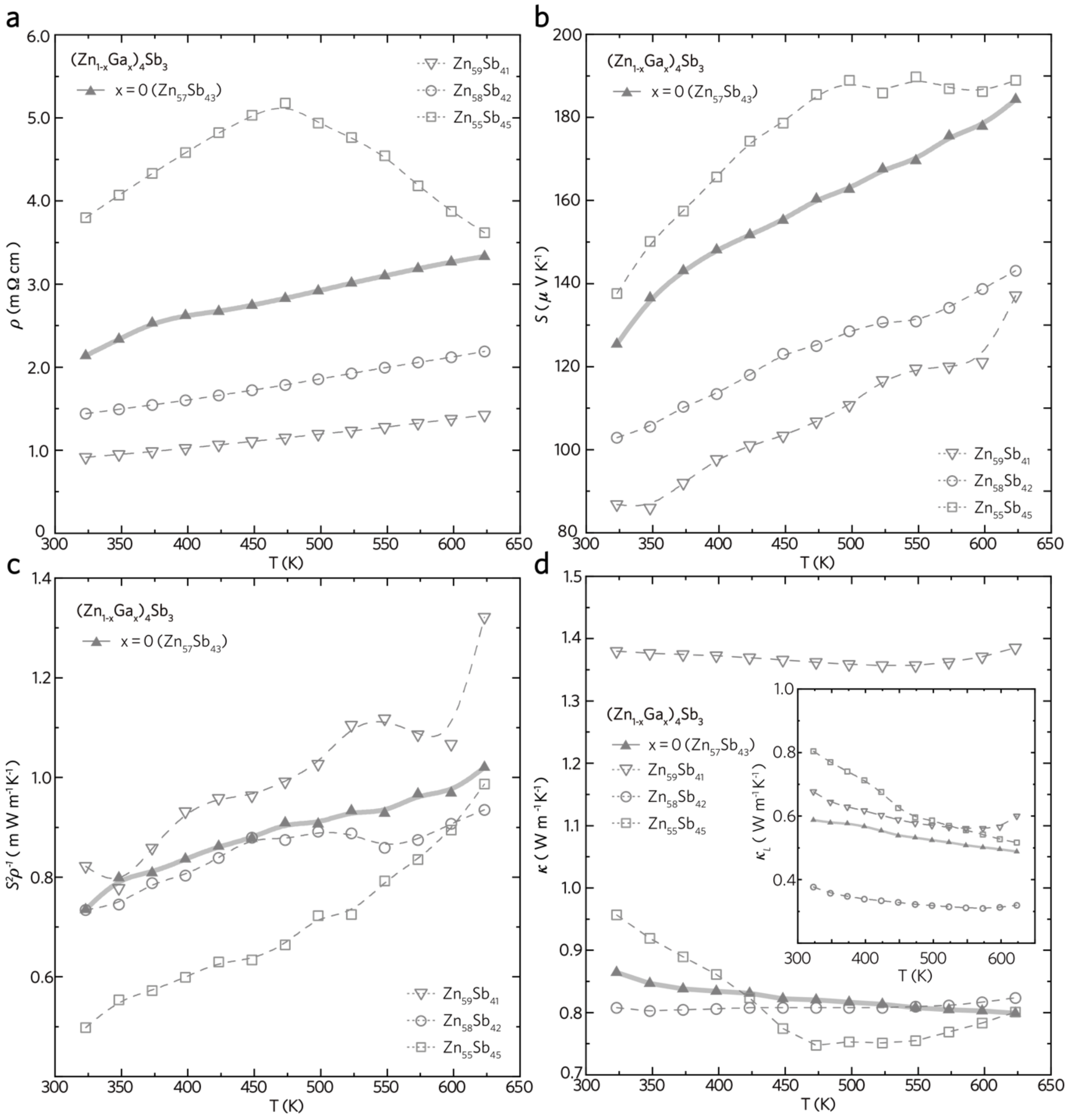

Figure S2. Temperature-dependent thermoelectric properties of Zn_zSb_{100-z} (z = 59, 58 and 55). a) Electrical resistivity, b) Seebeck coefficient *S*, c) power factor $S^2\rho^{-1,}$ and d) thermal conductivity *κ* from 320 K to 623 K. Inset shows lattice thermal conductivity κ_L.

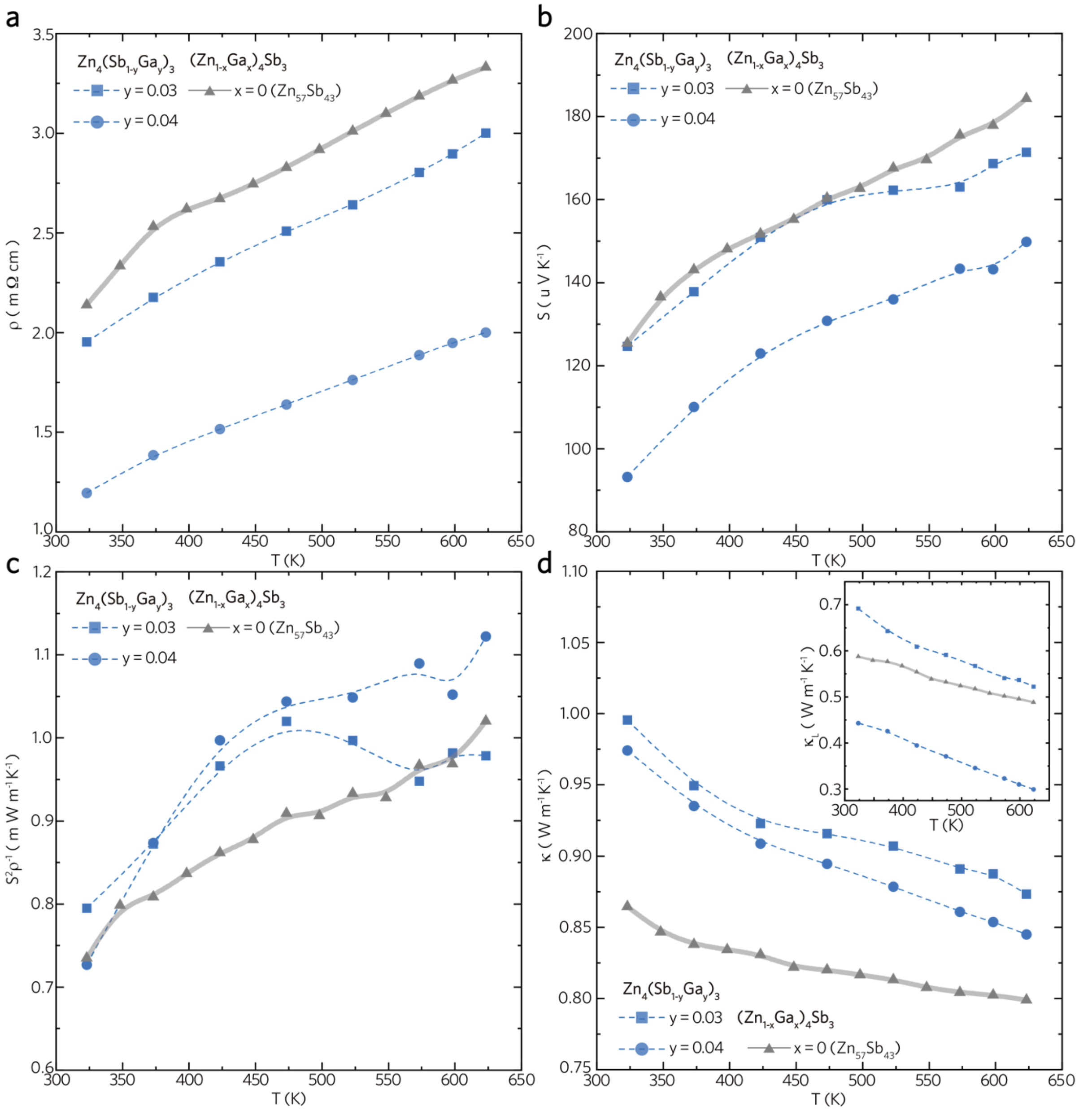

Figure S3. Temperature-dependent thermoelectric properties of $Zn_4(Sb_{1-y}Ga_y)_3$ (y = 0.03 and 0.04). a) Electrical resistivity, b) Seebeck coefficient S, c) power factor $S^2\rho^{-1}$, and d) thermal conductivity κ from 320 K to 623 K. Inset shows lattice thermal conductivity κ_L.

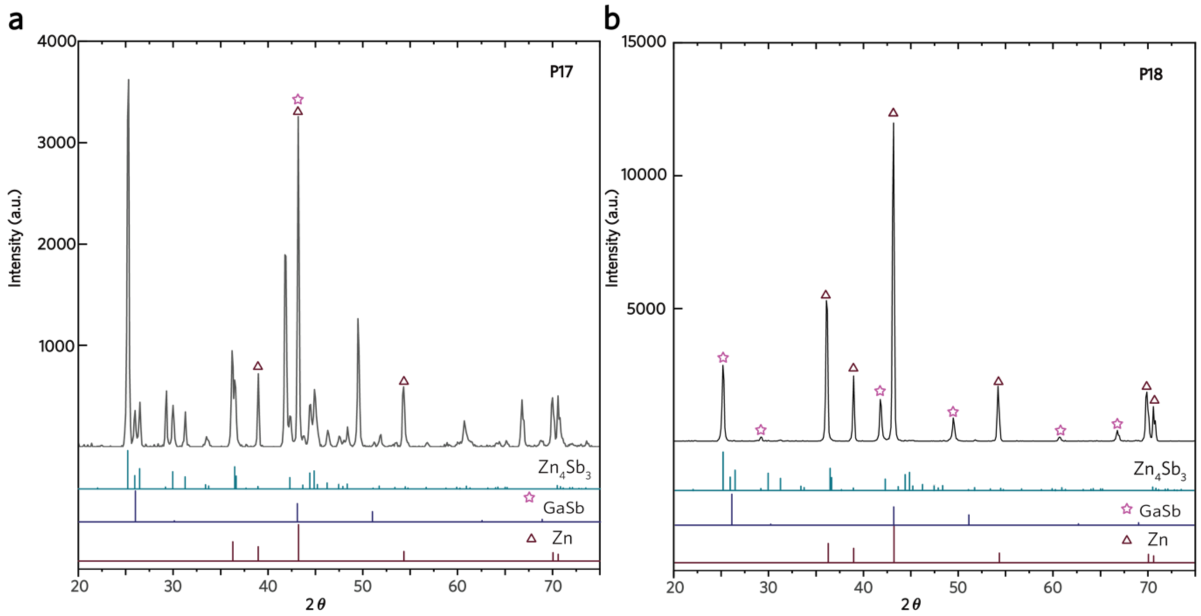

Figure S4. In-house XRD of Zn-Sb-Ga alloys equilibrated for over 60 days at 623 K: a) alloy #18 (Zn-5.0 at%Sb-5.0 at%Ga), b) alloy #17 (Zn-30.0 at%Sb-10.0at%Ga).

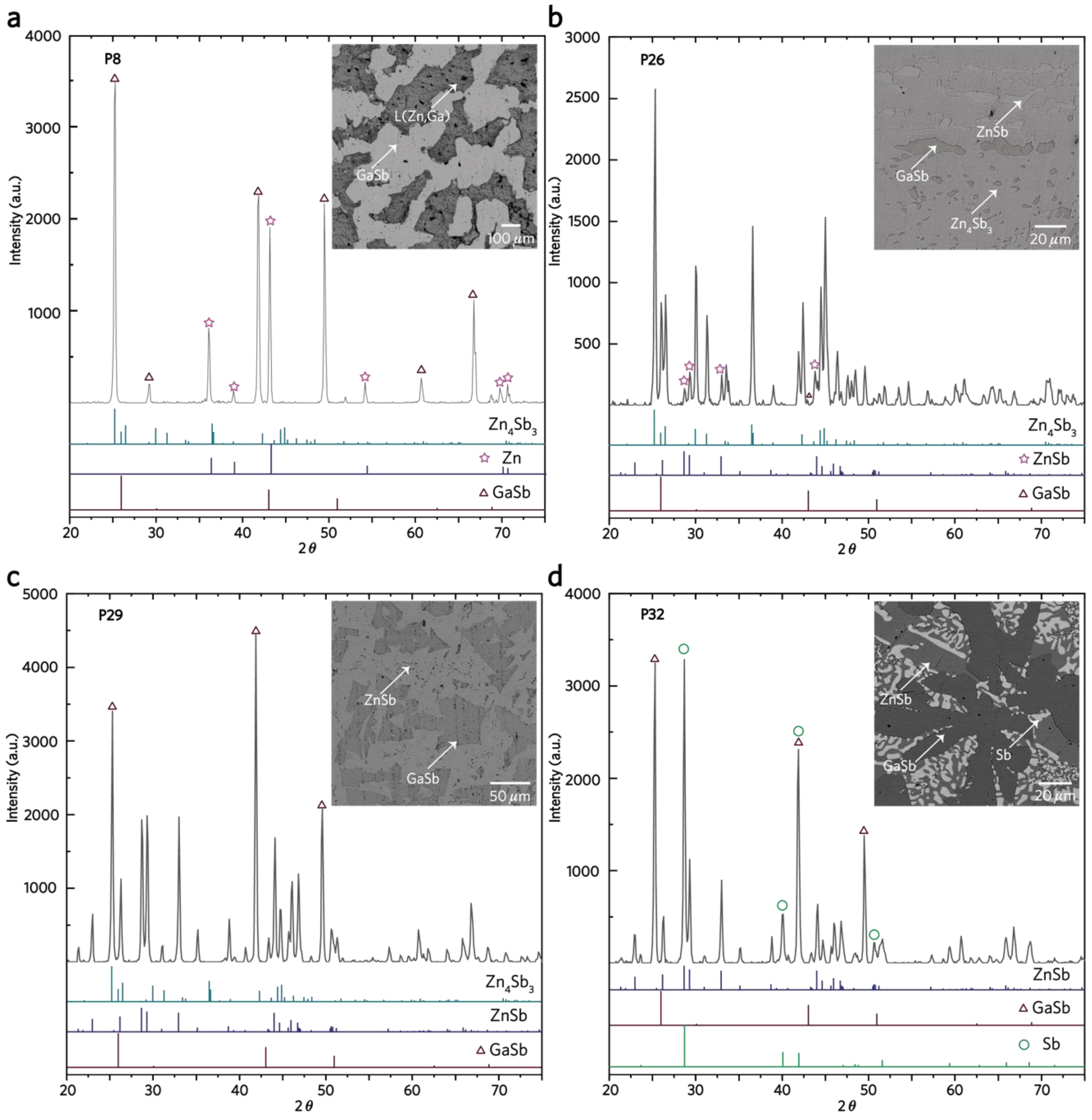

Figure S5. In-house XRD of Zn-Sb-Ga alloys equilibrated for over 60 days at 623 K: a) alloy #8 (Zn-20.0 at%Sb-30.0 at%Ga),b) alloy #26 (Zn-45.0 at%Sb-5.0 at%Ga), c) alloy #29 (Zn-50.0 at%Sb-15.0at%Ga), d) alloy #32 (Zn-60.0 at%Sb-15.0at%Ga). Inset shows the back scattered images.

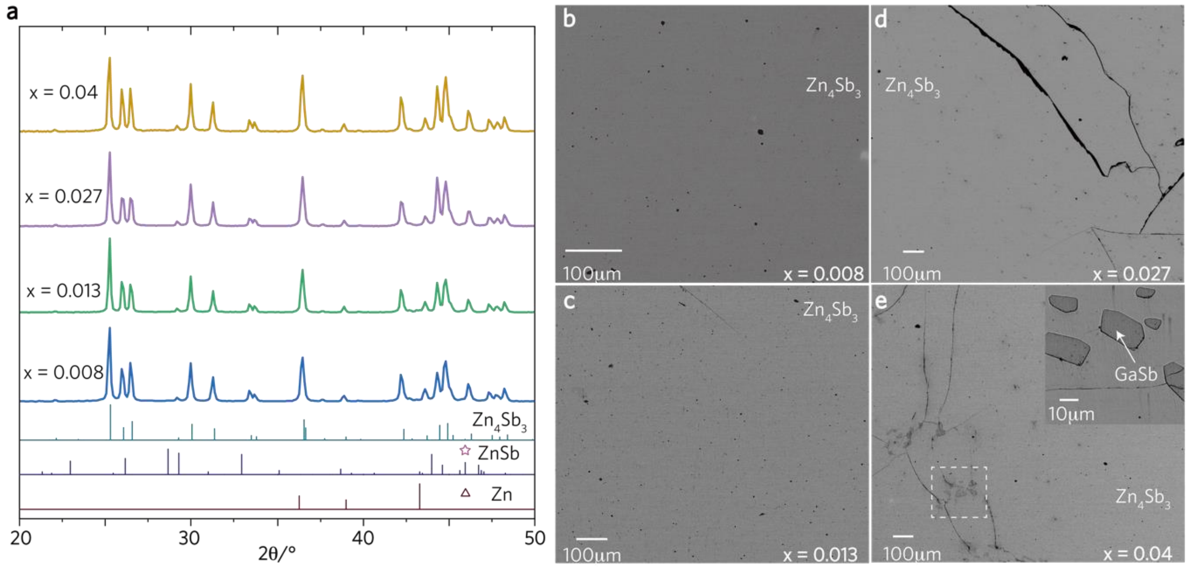

Figure S6. a) XRD patterns of Ga-Zn_4Sb_3 TE alloy for Zn_4Sb_3 and $(Zn_{1-x}Ga_x)_4Sb_3$ (x = 0.008, 0.013, 0.027 and 0.04), the back scattered images of Ga-Zn_4Sb_3 TE alloy: b) $(Zn_{0.995}Ga_{0.008})_4Sb_3$, c) $(Zn_{0.992}Ga_{0.013})_4Sb_3$, d) $(Zn_{0.973}Ga_{0.027})_4Sb_3$, e) $(Zn_{0.96}Ga_{0.04})_4Sb_3$.

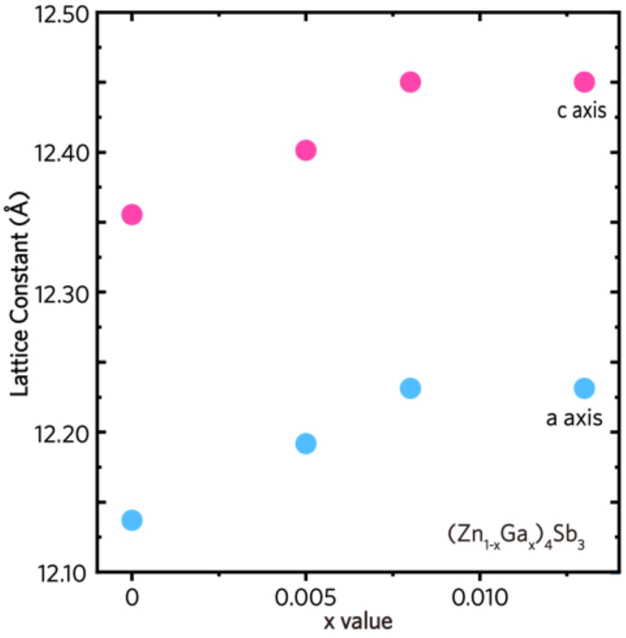

Figure S7. The lattice constants (*a* and *c* axis) of $(Zn_{1-x}Ga_x)_4Sb_3$ (x = 0, 0.005, 0.008 and 0.013) with increasing x. Red and blue dots show the *c* and *a* axis, respectively.

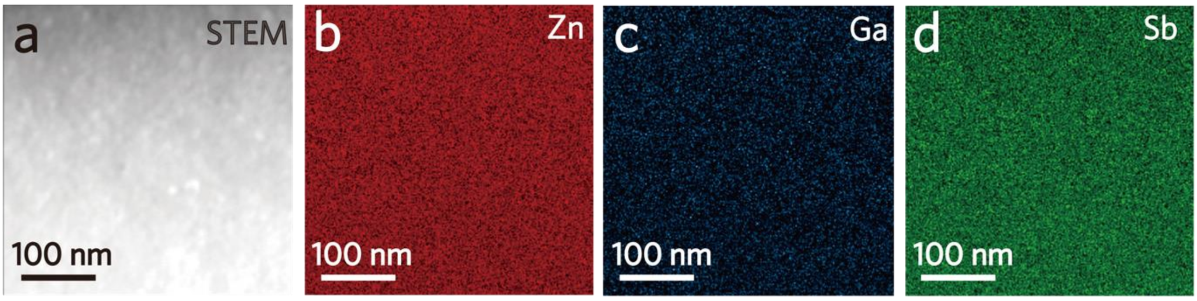

Figure S8. The STEM-EDS analyses of $(Zn_{0.992}Ga_{0.008})_4Sb_3$ alloy a-d) show the elemental mapping results.

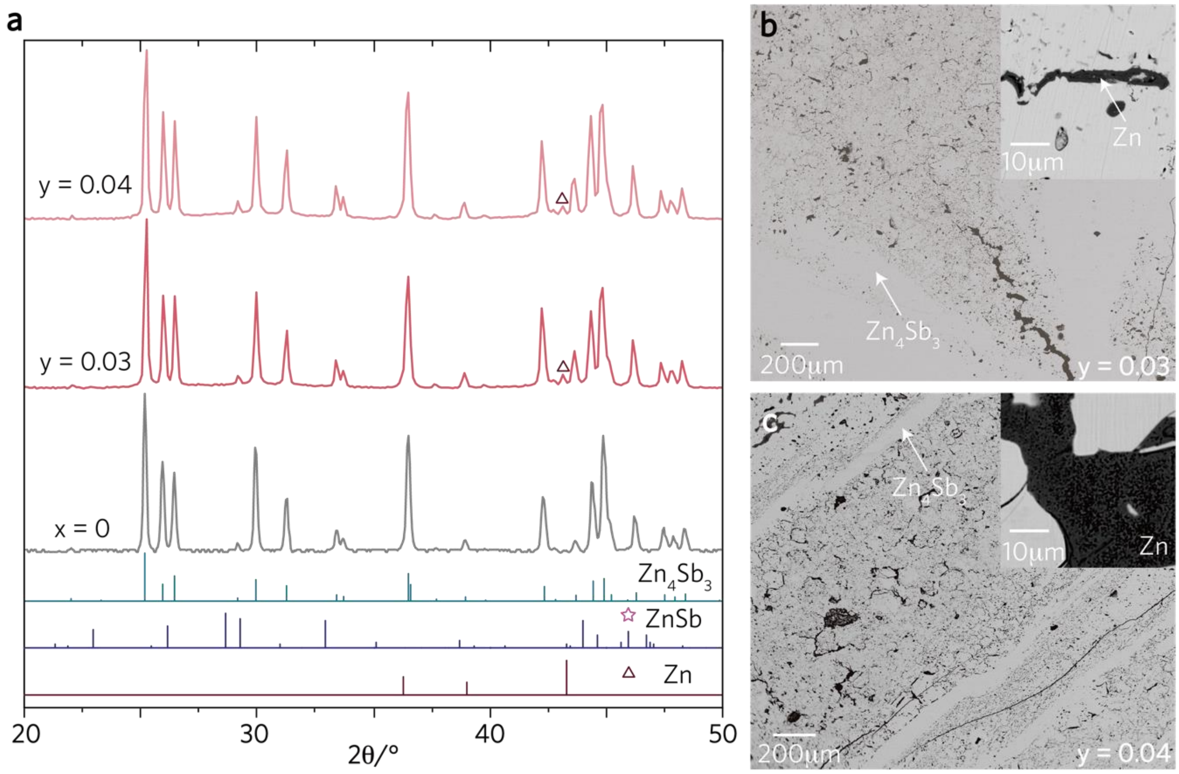

Figure S9. a) XRD patterns of Ga-Zn_4Sb_3 TE alloy for Zn_4Sb_3 and $Zn_4(Sb_{1-y}Ga_y)_3$ (y = 0.03 and 0.04), the backscattered images (BEI) of Ga-Zn_4Sb_3 TE alloy: b) $Zn_4(Sb_{0.97}Ga_{0.03})_3$, c) $Zn_4(Sb_{0.96}Ga_{0.04})_3$.

Table S1. WDS analysis of *x*-series alloys and binary Zn_zSb_{100-z} (z = 59, 58, and 55).

Alloy	Phase composition (at.%)			
	phase	Zn	Sb	Ga
Zn_4Sb_3	Zn_4Sb_3	56.9±0.6	43.1±0.6	0
x = 0.008	Zn_4Sb_3	56.0±0.7	43.8±0.8	0.3±0.2
x = 0.013	Zn_4Sb_3	56.2±0.9	43.5±0.8	0.3±0.1
x = 0.027	Zn_4Sb_3	54.3±0.3	44.5±0.3	1.3±0.1
$Zn_{55}Sb_{45}$	Zn_4Sb_3	56.9±0.3	43.1±0.3	0
	ZnSb	50.8±0.4	49.2±0.4	0
$Zn_{58}Sb_{42}$	Zn_4Sb_3	57.9±0.6	42.1±0.6	0
	Zn	100±0.0	0.0±0.0	0
$Zn_{59}Sb_{41}$	Zn_4Sb_3	56.9±0.5	43.1±0.5	0
	Zn	100±0.0	0.0±0.0	0

Table S2. Thermal and electrical transport properties of *x*-series alloys and binary Zn_zSb_{100-z} (z = 59, 58, and 55).

Compound	ρ [mΩcm]	S [μVK^{-1}]	κ [Wm^{-1}K^{-1}]	zT_{peak}	n_H [10^{19}cm^{-3}]	μ_H [cm^{2}V^{-1}s^{-1}]	d [g/cm^3]	*Relative Density* [%][9]
Zn_4Sb_3	2.14	125.4	0.86	0.80	2.84	103.1	6.18	99.5
x = 0.008	1.35	137.9	0.85	1.41	3.11	149.7	6.14	98.9
x = 0.013	1.44	122.3	0.91	1.28	6.21	70.1	6.11	98.3
x = 0.027	1.41	106.3	0.91	1.08	6.44	69.7	6.14	98.9
$Zn_{55}Sb_{45}$	1.69	137.6	0.96	0.77	2.60	145.1	6.14	98.9
$Zn_{58}Sb_{42}$	1.44	102.9	0.81	0.70	3.78	115.7	6.29	100.3
$Zn_{59}Sb_{41}$	0.92	86.8	1.38	0.59	5.91	116.1	6.13	98.7

Table S3. Nominal compositions of Zn-Sb-Ga ternary alloys equilibrated at 623 K together with the compositions of the equilibrium phases.

No.of Alloy	Nominal composition (at.%)			Phase composition (at.%)			
	Zn	Sb	Ga	phase	Zn	Sb	Ga
1	5	40	55	GaSb	1.6±0.6	51.4±0.6	47.0±0.9
				Liquid	3.3±0.0	0.4±0.0	96.3±0.0
2	20	30	50	GaSb	3.0±0.1	49.9±0.1	47.1±0.2
				Liquid	52.7±1.7	0.5±0.2	46.8±1.8
3	10	40	50	GaSb	2.4±0.0	51.4±0.0	46.2±0.0
				Liquid	1.9±0.0	0.1±0.0	98.0±0.0
4	10	45	40	GaSb	1.9±0.5	51.5±0.7	46.6±0.8
				Liquid	69.9±0.0	0.0±0.0	30.1±0.0
5	10	45	45	GaSb	2.5±0.9	51.5±0.3	46.0±0.3
				Liquid	62.9±0.0	0.2±0.0	36.9±0.0
6	30	30	40	GaSb	2.2±0.2	51.5±0.6	46.3±0.5
				Liquid	76.0±0.0	0.5±0.0	23.5±0.0
7	20	40	40	GaSb	2.8±0.8	51.6±0.5	45.6±0.6
				Liquid	92.1±0.0	0.3±0.0	7.6±0.0
8	50	20	30	GaSb	3.7±0.7	50.3±0.4	46.0±0.3
				Liquid	79.7±1.6	0.0±0.0	20.3±1.7
9	40	30	30	GaSb	5.1±0.5	50.4±0.9	44.5±1.4
				Liquid	96.6±0.0	0.1±0.0	3.3±0.0

10	77.5	2.5	20	GaSb	0.6±0.0	53.2±0.0	46.2±0.0
				Liquid	79.7±1.6	0.0±0.0	20.3±1.7
11	87.5	2.5	10	GaSb	2.4±0.0	48.9±0.0	48.7±0.0
				Liquid	89.1±0.0	0.0±0.0	10.9±0.0
12	60	20	20	GaSb	5.8±0.5	49.4±0.5	44.8±0.9
				Zn	95.9±0.6	0.6±0.4	3.5±0.2
13	80	10	10	GaSb	6.8±1.1	47.9±0.4	45.3±0.7
				Zn	98.5±0.5	0.3±0.4	1.2±0.2
14	92.5	2.5	5	GaSb	2.5±0.8	48.3±0.1	49.2±0.9
				Zn	98.9±0.1	0.0±0.0	1.1±0.1
15	50	30	20	GaSb	3.7±0.0	49.5±0.0	46.8±0.0
				Zn_4Sb_3	53.5±0.0	43.0±0.0	3.5±0.0
				Zn	97.9±0.9	1.3±0.7	0.8±0.5
16	70	20	10	GaSb	5.0±0.0	48.8±0.0	46.2±0.0
				Zn_4Sb_3	54.0±0.0	42.5±0.0	3.5±0.0
				Zn	99.1±0.2	0.1±0.0	0.8±0.1
17	60	30	10	GaSb	5.3±0.0	49.5±0.0	45.2±0.0
				Zn_4Sb_3	55.3±0.3	42.1±0.5	2.6±0.2
				Zn	98.8±0.5	0.1±0.0	1.1±0.5
18	90	5	5	GaSb	5.9±0.6	49.4±0.1	44.7±0.4
				Zn	98.8±0.5	0.1±0.0	1.1±0.5
19	85	10	5	GaSb	8.2±1.6	48.1±0.6	43.7±1.0

				Zn_4Sb_3	55.1±0.1	42.0±0.0	2.9±0.1
				Zn	99.4±0.0	0.0±0.0	0.6±0.0
				GaSb	4.3±0.0	51.6±0.0	44.2±0.0
20	80	15	5	Zn_4Sb_3	51.4±0.0	44.8±0.0	3.8±0.0
				Zn	99.1±0.5	0.12±0.1	0.8±0.4
				GaSb	5.8±0.1	49.8±0.0	44.4±0.2
21	75	20	5	Zn_4Sb_3	54.0±0.0	43.3±0.0	2.7±0.0
				Zn	99.0±0.4	0.5±0.3	0.5±0.1
				GaSb	4.3±0.0	49.2±0.0	46.5±0.0
22	75	20	5	Zn_4Sb_3	53.8±0.9	42.8±1.0	3.4±0.1
				Zn	99.4±0.0	0.2±0.0	0.4±0.0
				GaSb	6.9±1.0	49.7±0.3	43.4±0.8
23	65	30	5	Zn_4Sb_3	54.7±0.1	42.8±0.1	2.5±0.1
				Zn	99.8±0.0	0.2±0.0	0.0±0.0
				GaSb	6.9±0.0	50.2±0.0	42.9±0.0
24	92.5	5	2.5	Zn_4Sb_3	53.0±0.0	43.3±0.0	3.7±0.0
				Zn	99.5±0.0	0.0±0.0	0.5±0.0
				GaSb	5.5±0.1	50.8±0.3	43.7±0.4
25	40	45	15				
				Zn_4Sb_3	53.1±0.0	43.8±0.0	3.1±0.0
				GaSb	50.1±0.2	49.5±0.2	0.4±0.2
26	50	45	5	ZnSb	50.1±0.2	49.5±0.2	0.4±0.2
				Zn_4Sb_3	53.7±0.6	43.8±0.3	2.5±0.3

27	10	50	40	GaSb	1.2±0.3	52.1±0.5	46.7±0.3
				ZnSb	48.8±0.0	49.8±0.0	1.4±0.0
28	20	50	30	GaSb	1.5±0.2	51.7±0.3	46.8±0.5
				ZnSb	48.5±0.9	50.3±0.2	1.2±0.9
29	35	50	15	GaSb	2.5±0.0	51.5±0.1	46.0±0.1
				ZnSb	48.8±0.8	50.6±0.8	0.6±0.0
30	40	50	10	GaSb	2.7±0.2	50.5±0.3	46.8±0.4
				ZnSb	48.8±1.1	50.1±0.3	1.1±0.9
31	10	60	30	GaSb	2.0±0.3	51.5±0.6	46.5±0.7
				ZnSb	47.0±0.3	51.2±1.0	1.7±0.6
				Sb	1.50±0.1	96.3±0.7	2.2±0.8
32	25	60	15	GaSb	2.3±0.1	53.2±0.1	44.5±0.2
				ZnSb	47.4±0.0	51.5±0.0	1.1±0.0
				Sb	1.5±0.2	96.1±0.4	2.4±0.5
33	20	65	15	GaSb	2.0±0.0	52.4±0.6	45.6±0.6
				ZnSb	47.9±0.4	51.1±0.1	1.0±0.3
				Sb	1.1±0.1	97.9±0.0	1.0±0.2
34	10	80	10	GaSb	2.7±0.0	52.6±0.0	44.7±0.0
				ZnSb	50.1±1.3	49.5±1.3	0.4±0.0
				Sb	0.7±0.3	98.8±0.3	0.5±0.0
